# Hematopoietic JAK2^V617F^-mediated clonal hematopoiesis: AIM2 understand mechanisms of atherogenesis

**DOI:** 10.20517/jca.2021.06

**Published:** 2021-06-06

**Authors:** Soichi Sano, Kenneth Walsh

**Affiliations:** 1 Department of Cardiology, Osaka City University Graduate School of Medicine, Osaka 558-8585, Japan; 2 Hematovascular Biology Center, Robert M, Berne Cardiovascular Research Center, University of Virginia School of Medicine, Charlottesville, VA 22908, USA

The age-dependent accumulation of somatic DNA mutations in “driver” genes within the hematopoietic system can provide a fitness advantage to the mutant cells and thus allow for their aberrant clonal expansion, a process known as clonal hematopoiesis (CH). While this process is clearly recognized to initiate cancers in the hematopoietic system, numerous studies have documented that this condition is remarkably widespread in the general population and that individuals who harbor these mutations rarely go on to develop a hematologic malignancy. Thus, this condition has been referred to as clonal hematopoiesis of indeterminate potential or age-related clonal hematopoiesis. Multiple lines of evidence indicate that this prevalent form of CH is associated with higher rates of mortality, and cause-specific analyses indicate that CH resulting from mutations in any one of a number of driver genes will increase the incidence of coronary heart disease and ischemic stroke. Individuals with clonal hematopoiesis due to the *JAK2^V617F^* driver mutation are reported to have a 12-times higher risk for myocardial infarction, while other major CH driver gene mutations (e.g., *TET2*, *DNMT3A*, *ASXL1*) increased the risk by 1.7–2.0-fold^[1]^. Additionally, it has been reported that *JAK2^V617F^*-mediated CH increases the risk of developing venous thrombosis^[2]^. Therefore, individuals with the *JAK2^V617F^* mutation in hematopoietic cells may be at a particularly high risk of developing cardiovascular disease (CVD).

The *JAK2^V617F^* mutation, which leads to the constitutive activation of JAK2-associated cytokine receptor signaling, was originally recognized as being frequently mutated in myeloproliferative neoplasms (MPN) such as polycythemia vera and essential thrombocytosis. However, it has been reported that the *JAK2^V617F^* mutation can be detected at a low allele fraction in as many as 3.1% of the general population using a highly sensitive ddPCR methodology^[3]^. Thus, the apparent widespread detection of *JAK2^V617F^*-mediated clonal hematopoiesis has led to interest in experimental studies to elucidate the mechanisms by which the *JAK2^V617F^* mutation in hematopoietic cells can contribute to CVD.

In a previous study, this group reported that pan-hematopoietic *Jak2^V617F^* mutation (the *Jak2* mutation in mice is relevant to human *JAK2^V617F^*) promotes atherogenesis in hyperlipidemic mice^[4]^. Specifically, the analysis of plaque composition revealed that the hematopoietic *Jak2* mutation increases neutrophil infiltration in early atherosclerotic lesions and a larger necrotic core in the more advanced lesion, consistent with enhanced rolling and adhesion capacity of *Jak2*-mutant neutrophils (see Figure 1). Increased erythrophagocytosis and decreased efferocytosis by macrophages within the lesion were also observed in mice with the *Jak2^V617F^* mutation. In addition, red blood cells, either mouse or human, were more prone to erythrophagocytosis, presumably because of decreased expression of “don’t-eat-me” signals. Collectively, these results highlighted numerous possible mechanisms through which *JAK2^V617F^*-mediated CH could increase the risk of atherosclerotic CVD. However, a significant limitation of this study is that the pan-hematopoietic *Jak2^V617F^* mouse model displays MPN phenotypes, including increased hematocrit or platelet counts, that are not observed in individuals with CH. Thus, more refined models of *JAK2^V617F^*-mediated CH are required to more rigorously understand the nature of the CVD mechanisms.

The creation of a model of *JAK2-CH* is not straightforward because multiple mouse lines that harbor the *Jak2^V617F^* mutation in their hematopoietic stem and progenitor cells (HSPC) exhibit MPN phenotypes that confound the CVD phenotype under investigation. Thus, a strategy to the model of *JAK2-CH* is to establish mice that express the mutant *JAK2/Jak2* gene in specific blood cell populations such as neutrophils or monocytes/macrophages and potentially avoid confounding MPN phenotypes. In this regard, Sano *et al.*^[5]^ used a lentivirus vector encoding human *JAK2^V617F^* under myeloid-specific synthetic SP146 promoter/gp91 enhancer combination. This system avoids using the *Lyz2-Cre* mice to express the mutant transgene because this system is “leaky” and leads to low *JAK2^V617F^* expression in HSPC and the MPN-associated expansion of mutant blood cells. In contrast, bone marrow reconstruction of lethally irradiated mice with HSPC transduced with the lentivirus construct allowed *JAK2^V617F^* to be expressed only in myeloid cell lineages, and this avoids the MPN-like hematological abnormalities. These myeloid-specific *JAK2^V617F^* mice exhibited accelerated cardiac dysfunction following experimental myocardial infarction or pressure overload-mediated hypertrophy. Moreover, these heart failure phenotypes were associated with cytokine activation, including IL-1β, in the myocardium.

In the more recent study, Fidler *et al*.^[6]^ utilized *Cre/LoxP*-mediated recombination to achieve *Jak2^V617F^* expression in specific myeloid cell types. Specifically, *S100a8-Cre* and *Cx3cr1-Cre* mice were employed to achieve neutrophil- and monocyte/macrophage-specific *Jak2^V617F^* expression, respectively (see Figure 1). Similar to the earlier findings^[5]^, neither of these strains displayed MPN characteristics, thereby allowing the analysis of CVD phenotypes of *Jak2^V617F^* in each myeloid population in the absence of elevated blood cell counts. It was found that, hyperlipidemic mice reconstituted with bone marrow cells from *Cx3cr1-Cre/Jak2^V617F^* mice, exhibited increased atherosclerotic plaque size and signs of vulnerable plaque despite lower serum low-density-lipoprotein cholesterol and triglyceride levels^[6]^. Consistent with larger necrotic core, plaques in the *Cx3cr1-Cre/Jak2^V617F^* mice also contained greater numbers of macrophages due to a higher proliferation rate. On the other hand, mice with bone marrow cells from *S100a8-Cre/Jak2^V617F^* mice did not display increased atherosclerosis progression. This finding is somewhat unexpected, considering the previous report that *Jak2^V617F^* increases neutrophil rolling and adhesion to vascular wall^[4]^ and the current understanding that neutrophils are associated with atherogenesis^[7]^. Regardless, these analyses highlight the importance of rigorous cell type-specific expression analyses to delineate the primary cell types that mediate the atherogenic effects of CH.

Fidler *et al*.^[6]^ reported that *Jak2^V617F^* macrophages express higher levels of the AIM2 inflammasome. Similarly, increased *AIM2* expression has been observed in *JAK2^V617F^*-expressing human monocyte/macrophage THP-1 cells^[5]^. Further analyses revealed that AIM2 is regulated by elevated IFNg signaling which, in turn, is activated by increased oxidative DNA damage and replication stress^[6]^. The activation of AIM2 leads to the over-production of IL-1β and IL-18 and also promotes Gasdermin-mediated pyroptosis. Notably, it was shown that deficiency of the AIM2 inflammasome or inhibition of IL-1β signaling largely reversed the atherosclerosis phenotype driven by *Jak2^V617F^* in hematopoietic cells, whereas deficiency of the NLRP3 inflammasome did not show protection from atherosclerosis under these conditions. In contrast, pharmacological inhibition of the NLRP3 inflammasome inhibited atherogenesis and heart failure in models of mutant *Tet2*-mediated clonal hematopoiesis^[8,9]^. Collectively, these data raise the exciting possibility of mutant gene-specific differences in how CH modulates CVD. Thus, by extension of these findings, there may be a rationale for personalized therapies based upon the diagnosis of the specific gene(s) mutated in CH.

*JAK2^V617F^*-mutant HSPC can give rise to multiple lineages of blood cells including myeloid cells, platelets, red blood cells, *etc*. Thus, the development of CVD models with selective *Jak2^V617F^* expression in additional blood cell lineages is warranted. A previous study has shown that *Jak2^V617F^*-mutant neutrophils display increased neutrophil extracellular trap (NET) formation in both humans and mice, which is associated with venous thrombosis formation^[2]^. As discussed, *Jak2^V617F^*-mutant macrophages, but not neutrophils, are contribute to enhanced atherogenesis through an AIM2 inflammasome-dependent mechanism^[7]^. Given these findings, it would be of interest to assess whether *JAK2-CH* is enriched in patients with plaque erosion, as this form of CVD event is associated with NET formation. Finally, it would also be of interest to examine the pathogenic roles of *JAK2^V617F^* in other blood cell types using selective expression strategies in mice.

## Figures and Tables

**Figure 1.**
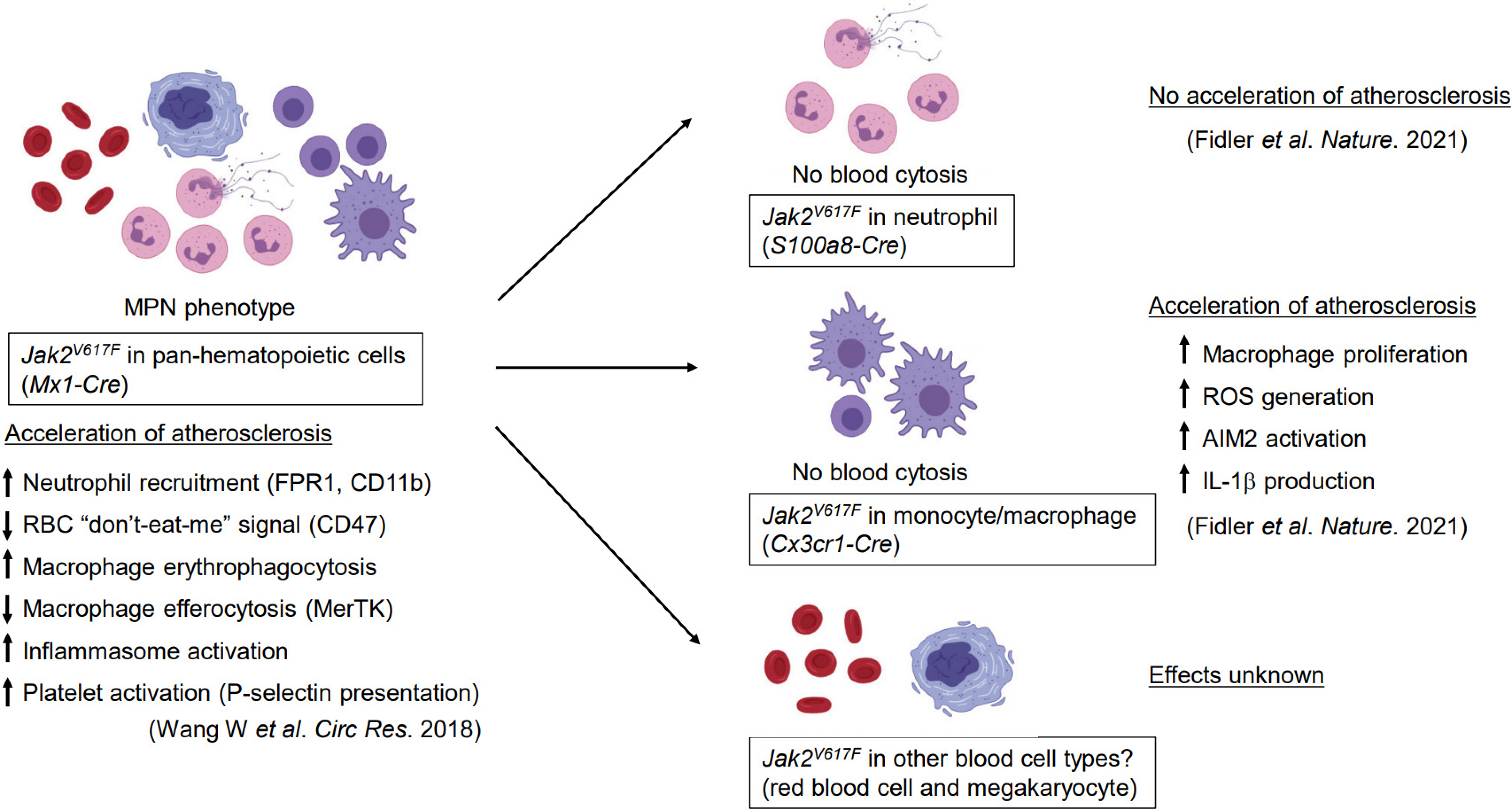
Experimental studies of the *Jak2V617F* mutation in blood and its effects on atherogenesis.
